# The stability of carbapenems before and after admixture to PMMA-cement used for replacement surgery caused by Gram-negative bacteria

**DOI:** 10.1186/s40001-020-00428-z

**Published:** 2020-08-18

**Authors:** Matthias Schmid, Oliver Steiner, Lisa Fasshold, Walter Goessler, Anna-Maria Holl, Klaus-Dieter Kühn

**Affiliations:** 1 Department of Orthopaedics and Orthopaedic Surgery, Medical University Graz, Auenbruggerplatz 34, 8036 Graz, Austria; 2 Institute of Chemistry, Analytical Chemistry for Health and Environment, University of Graz, Universitätsplatz 1, 8010 Graz, Austria; 3 Clinical Affairs, Heraeus Medical GmbH, Philipp-Reis-Straße 8-13, 61273 Wehrheim, Germany

**Keywords:** Imipenem, Meropenem, Carbapenems, PMMA cement, Periprosthetic joint infection, Local antibiotic therapy, Spacer, Antibiotic-loaded bone cement

## Abstract

**Background:**

Prosthetic joint infection (PJI) is a serious complication of orthopedic implant surgery. Treatment often includes the use of an antibiotic-loaded Polymethyl methacrylate (PMMA) bone cement spacer. Several antibiotics are commonly used for the preparation of these spacers, but due to the increasing number of infections with resistant Gram-negative bacteria, there is a need for the use of carbapenem antibiotics such as meropenem and imipenem as drugs of last resort. Unfortunately, the reaction heat generated during the preparation of the bone cement can be a major problem for the stability of these antibiotics. In the present study, the stability of meropenem and imipenem was tested before and after the admixture to PMMA bone cements.

**Methods:**

High-performance liquid chromatography with ion-pairing reversed-phase separation and spectrophotometric detection was used for analysis. Stability tests with meropenem and imipenem were performed with antibiotics in solution and solid form at different temperatures (37 °C, 45 °C, 60 °C, 90 °C) and times (30 min, 60 min, 120 min). To test the stability of both antibiotics in PMMA after exposure to the reaction heat during polymerization, three different bone cements were used to generate specimens that contained defined amounts of antibiotics. Reaction heat was measured. The form bodies were mechanically crushed and aliquots were dissolved in ethyl acetate. Samples were prepared for HPLC DAD analysis.

**Results:**

Meropenem and imipenem showed the highest degradation levels after heat stressed in solution, with maximum levels of 75% and 95%, respectively. In solid form, degradation levels decreased dramatically for meropenem (5%) and imipenem (13%). Stability tests of both carbapenems in bone cement showed that they remained largely stable during PMMA polymerization, with retrieved amounts of about 70% in Palacos^®^ R and Copal^®^ G+V, and between 80 and 90% in Copal^®^ spacem.

**Conclusions:**

In contrast to the results of meropenem and imipenem in solution, both antibiotics remain stable in solid form and mostly stable in the cement after PMMA polymerization. The low degradation levels of both antibiotics after exposure to temperatures > 100 °C allow the conclusion that they can potentially be used for an application in PMMA cements.

## Background

Arthrosis deformans (Osteoarthritis, OA) is a degenerative disease that represents a major challenge for surgical treatment. Surgical treatment of OA mostly consists of joint replacement. The number of joint replacement arthroplasties increased in many countries in recent years [1, 2]. Operative joint replacements are associated with many risk factors, among which joint infection is a feared and serious complication in joint arthroplasty as it is most difficult to treat.

A major problem is biofilm formation on the implant surface. Compared to planktonic germs, the efficacy of antibiotics toward sessile germs is considerably reduced [3]. A biofilm is established by the conglomeration of bacterial cells that attach to the implant surface and produce a protective extracellular matrix. Biofilms are estimated to provoke about 65% of human infections [4, 5]. Several approaches for surgical treatment of joint infection have been described [6, 7].

**Fig. 1:**
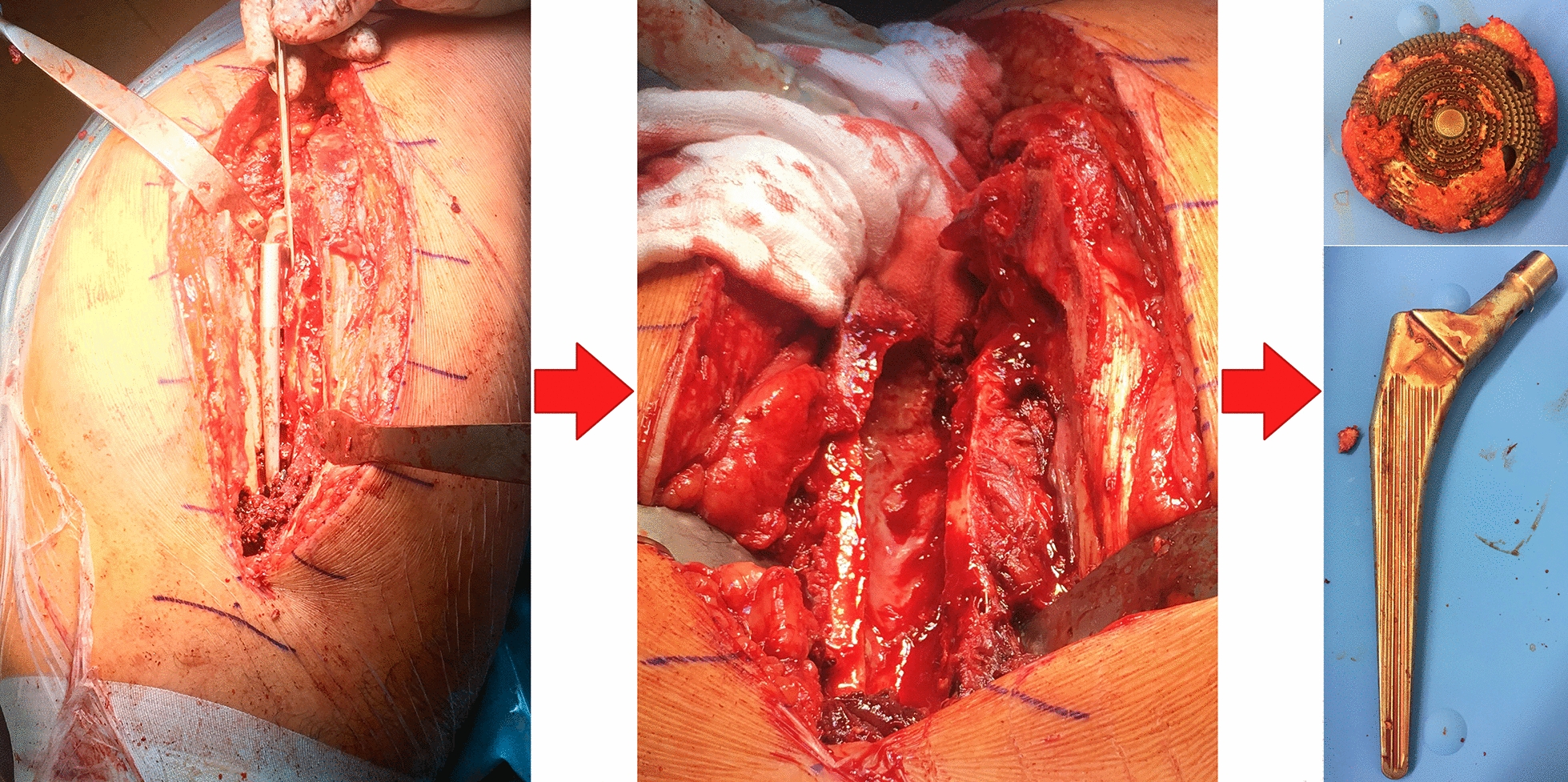
Explantation of an infected hip implant in the context of a two-stage revision

Treatment includes one-stage revision, two-stage revision or three-stage revision of the infected implant, along with a radical debridement. Two-stage revision is the gold standard and can be performed with short or long interval [8]. Short interval means removal of the infected prosthesis and reassembly of a new implant after 2 weeks. Long interval includes the implantation of a PMMA (Polymethyl methacrylate) bone cement spacer to bypass the interval between explantation of the old and implantation of the new prosthesis (Fig. 1) [8]. Bone cements can be deployed for local antibiotic therapy. Treatment success depends on the quality of pathogens and their resistance to antibiotics [9].

For local antibiotic therapy, the PMMA bone cement spacer is loaded with appropriate antibiotic(s). With this method, high local antibiotic concentrations can be obtained at the spacer surface for efficient infection treatment. A pathogen eradication of more than 90% after two-stage revision has been reported [10]. Gentamicin, clindamycin, tobramycin, vancomycin, erythromycin and colistin represent antibiotics currently used in bone cements for surgical therapy, as they are heat resistant and long lasting [11]. Due to the rising number of multi-resistant bacteria, recent publications recommend the addition of other antibiotics for local treatment [11, 12]. To retain the stability, a maximum content of 10% antibiotics in bone cement should not be exceeded [11].

Increasing numbers of infections with multi-resistant Gram-negative bacteria, such as *Pseudomas aeruginosa* or *Enterobactericeae*, make it necessary to consult carbapenems for local and systemic antibiosis [11]. Carbapenems are antibiotics of last resort and belong to β-lactams with high resistance against β-lactamases. They are able to eradicate a very broad spectrum of aerobic or anaerobic Gram-negative and Gram-positive bacteria [13, 14]. Both can be used to treat multi-resistant Gram-negative organisms, with imipenem being more active against Gram-positive cocci and meropenem against Gram-negative bacilli [13, 15–17]. Both belong to the carbapenem family and are mentioned in literature as potential candidates for antibiotic treatment of joint infections [12, 18]. Imipenem is usually combined with an equal amount of cilastatin, which is an inhibitor of dehydropeptidase-I and reduces the degradation of imipenem in renal tubules [19].

For the reasons mentioned above, meropenem and imipenem were chosen for the present tests. Cilastatin was not included in the analysis as the degradation via dehydropeptidase-I should not be relevant for a local treatment at the PMMA surface.

During the PMMA bone cement preparation, liquid monomer is mixed with the polymer powder component, generating high temperatures because of the radical initiated exothermic polymerization reaction. This represents a major problem for the used, heat-sensitive antibiotics [20].

Therefore, the stability of meropenem and imipenem is evaluated at different temperatures before and after admixture to PMMA cement in this study. We hypothesize an acceptable stability of the tested antibiotics in PMMA.

## Methods

The aim of the study was to test the stability of meropenem and imipenem before and after the admixture to PMMA bone cements.

### Stability of antibiotics before admixture to bone cement

Both antibiotics (Meropenem Kabi^®^, Imipenem/Cilastatin Kabi^®^, powders) were temperature stressed at 37 °C, 45 °C, 65 °C and 90 °C in solution (1 mg/mL, 0.9% NaCl) and solid phase to investigate possible thermal degradation. Samples of the heated antibiotics in powder or dissolved form were taken after 30, 60 and 120 min, and prepared for analysis with high-performance liquid chromatography with spectrophotometric detection (HPLC DAD). Degradation levels were analyzed by comparison with standards that contained 1 mg/mL of meropenem or imipenem. For every stability test, five replicates were prepared, containing either 5 mg of meropenem or imipenem/cilastatin.

### Stability of antibiotics after admixture to PMMA bone cement

The stability of both antibiotics was tested after exposure to the reaction heat generated during PMMA polymerization. The powder of three different PMMA cements (Palacos^®^ R, Copal^®^ G + V and Copal^®^ spacem; Heraeus Medical, Wehrheim) was mixed with meropenem or imipenem and polymerized according to operating instructions in standardized cylindrical form bodies (diameter: 25 mm, height: 12 mm, ~ 5 g). The mixture was performed according to a proven scheme [12]. The temperature was measured during the exothermic polymerization reaction for each specimen.

**Fig. 2:**
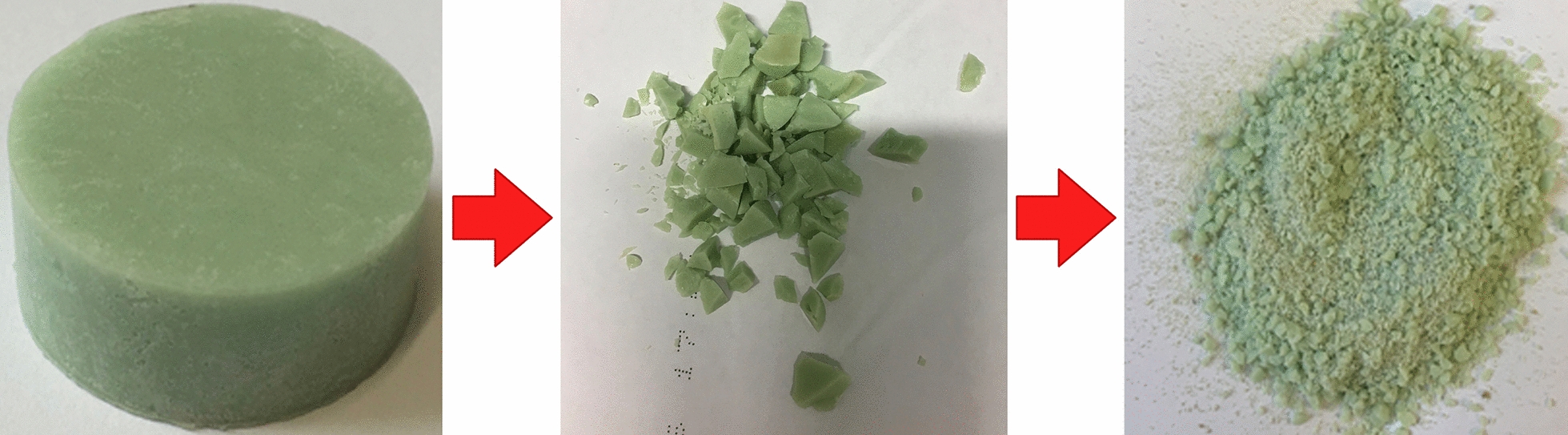
Crushing and grinding of bone cement form previously formed bodies

**Fig. 3:**
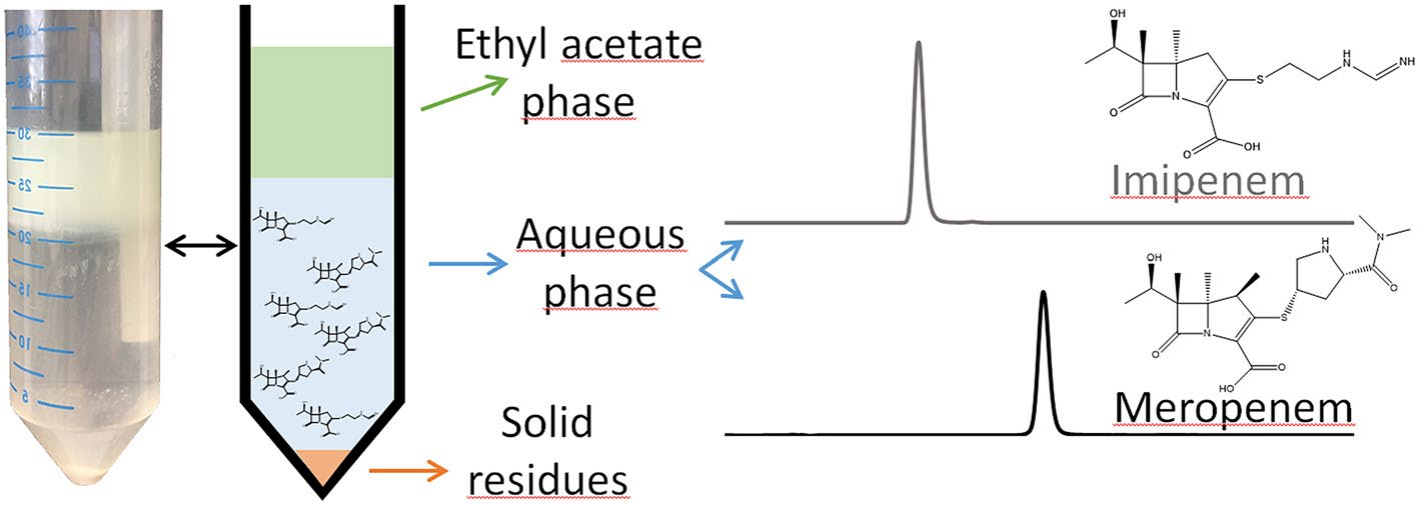
Tube with two phases, ethyl acetate phase with polymer, aqueous phase with dissolved antibiotics

The antibiotic concentration was selected to be 250 mg of antibiotic in 15 g (10 g polymer, 5 g monomer) PMMA bone cement (~ 1.6%). Four replicate samples were prepared for Palacos^®^ R, triplicates for Copal^®^ G + V and a single sample for Copal^®^ spacem. Another measurement was performed with an amount of 500 mg of meropenem or imipenem in 30 g Copal^®^ spacem cement (20 g polymer, 10 g monomer) to investigate the stability of the carbapenems at higher temperatures (used also for temperature measurements). The form bodies were mechanically crushed and grinded to maintain a powder for faster dissolution of the polymer including the antibiotics (Fig. 2). Aliquots of this powder (500 mg) were dissolved in ethyl acetate (10 mL), which turned out to be the most suitable solvent in pretests. Ethyl acetate as extraction solvent is advantageous compared to the other tested solvents (acetone, tetrahydrofuran, and chloroform). It achieves a good phase separation, shows a good partition coefficient and no interferences are observed during HPLC DAD analysis. The solution was vigorously mixed with ultrapure water (20 mL) and afterward centrifuged. The water and ethyl acetate phase were separated, resulting in an ethyl acetate phase containing the dissolved polymer, an aqueous phase that contained the dissolved antibiotics, and the insoluble filling and contrast materials (zirconia, X-ray contrast mediums) (Fig. 3).

### HPLC DAD methods

The concentration of meropenem and imipenem was determined by high-performance liquid chromatography with ion-pairing reversed-phase separation and spectrophotometric detection (IPRP-HPLC DAD). The HPLC DAD methods for meropenem and imipenem are given (Table 1).

**Table 1:** IPRP-HPLC DAD methods for meropenem and imipenem determination

Parameter	Meropenem method	Imipenem method
Flow rate [mL/min]	1.5	1
Mobile phase	75% of A and 25% of B	100% of B
Injection volume [µL]	1	
Detection wavelength	300 nm	
Autosampler temperature [°C]	5	
Column temperature [°C]	45	
Runtime [min]	4	

Mobile phase A was prepared by dissolving 12 g of tetrabutylammonium hydroxide in 780 mL of ultrapure water and mixing with 150 mL of acetonitrile and 70 mL of methanol. For mobile phase B, 12 g of tetrabutylammonium hydroxide was dissolved in 1000 mL ultrapure water. The pH of both mobile phases was adjusted to 7.5 with phosphoric acid.

An Agilent Zorbax Eclipse XDB—C18 50 × 4.6 mm, 1.8-µm particles’ column was used for both HPLC DAD methods.

### Temperature measurement

**Fig. 4:**
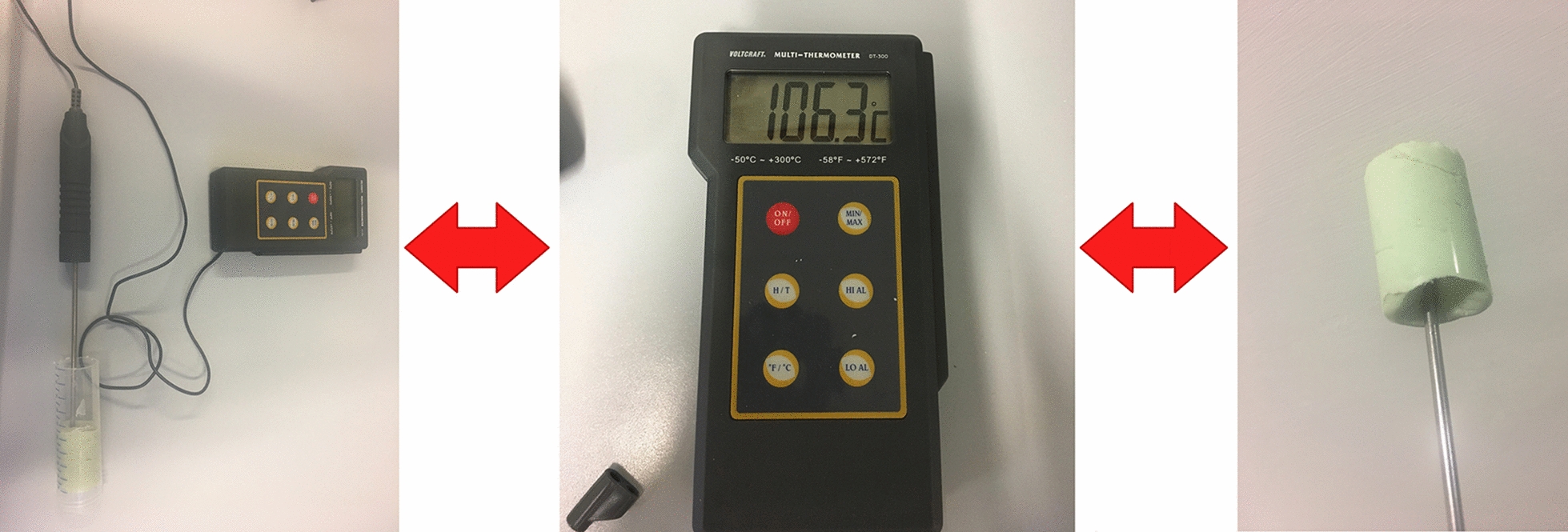
Temperature measurement experiment setup

The temperature measurement during polymerization of PMMA bone cement was performed with a Voltcraft DT 300 thermometer. Copal^®^ spacem, Copal^®^ G + V and Palacos^®^ R (30 g) cements were prepared. After preparation, the paste was transferred to a 50-mL polypropylene vessel, which is open at both sides. The measuring sensor was placed into the center of the bone cement mass (Fig. 4). The temperature was measured until 20 min after combining liquid monomer and powdery polymer. The resulting bone cement was crushed, dissolved, and submitted to HPLC DAD analysis as described above.

## Results

### Stability of antibiotics in solution

**Fig. 5:**
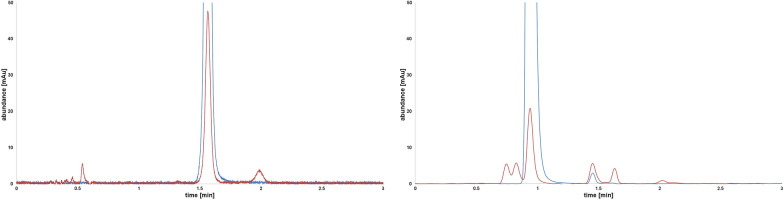
Chromatograms of meropenem (left) and imipenem (right). Standard (blue) and sample after 120 min at 90 °C (red), with occurring degradation products after thermal treatment

An increased degradation of meropenem and imipenem was observed in solution only at higher temperatures. Typical chromatograms of degraded meropenem and imipenem samples are depicted in Fig. 5.

**Fig. 6:**
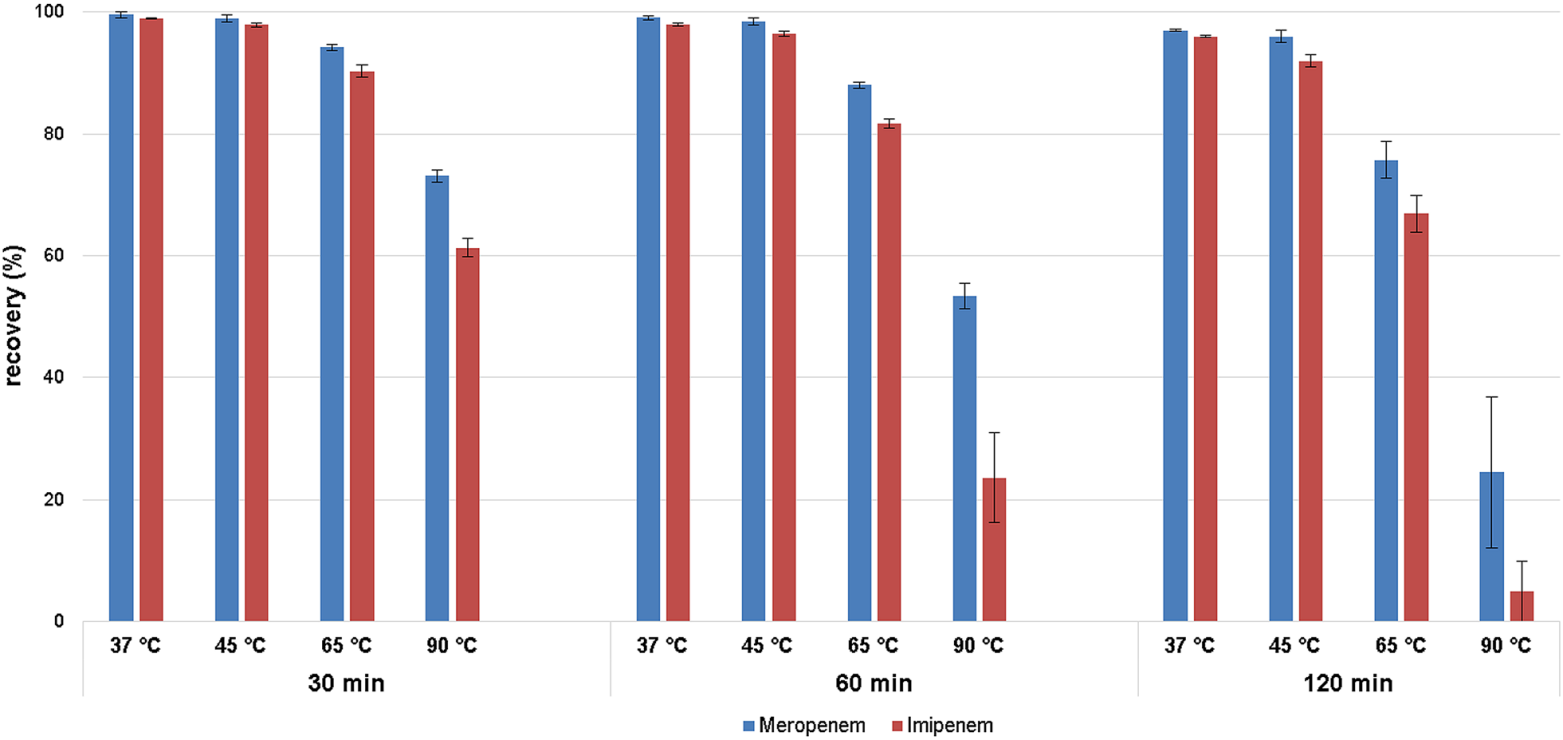
Recovery of temperature stressed meropenem/imipenem in solution after 30, 60, and 120 min (*n* = 5)

Figure 6 shows the recovery of temperature stressed meropenem and imipenem in solution at different times. The degradation levels of both antibiotics correlate with the heating time. Meropenem showed a degradation of 3% at 37 °C and 4% at 45 °C after 120 min, 25% at 65 °C and 75% at 90 °C. Imipenem showed a degradation of 4% at 37 °C and 8% at 45 °C after 120 min. Again, the degradation level increased with rising temperatures to 33% at 65 °C and even 95% at 90 °C.

Samples of meropenem or imipenem incubated for 30 or 60 min in solution showed lower degradation levels. Degradation of meropenem heated for 30 min was only 0.4% at 37 °C and 1% at 45 °C. Higher temperatures resulted in major destruction of meropenem. Degradation of meropenem at 65 °C and 90 °C was 5.8% and 27%, respectively.

Degradation levels of imipenem exposed to heat stress for 30 min were even higher. At 37 °C and 45 °C, the amount was 1% and 2.1%, respectively. Increasing temperatures led to an augmented degradation of imipenem, with 9.7% at 65 °C, and 39% at 90 °C.

After 60 min of heating, degradation of meropenem was 0.7% at 37 °C and 1.5% at 45 °C. Higher temperatures resulted in a degradation of 12% at 65 °C and 47% at 90 °C. Degradation levels of imipenem at 37 °C and 45 °C were 2% and 3.6%. Degradation increased at exalted temperatures. Imipenem showed a degradation of 19% at 65 °C and 76% at 90 °C.

Overall, heat-stressed meropenem showed higher stability compared to imipenem in solution.

### Stability of antibiotics in solid phase

**Fig. 7:**
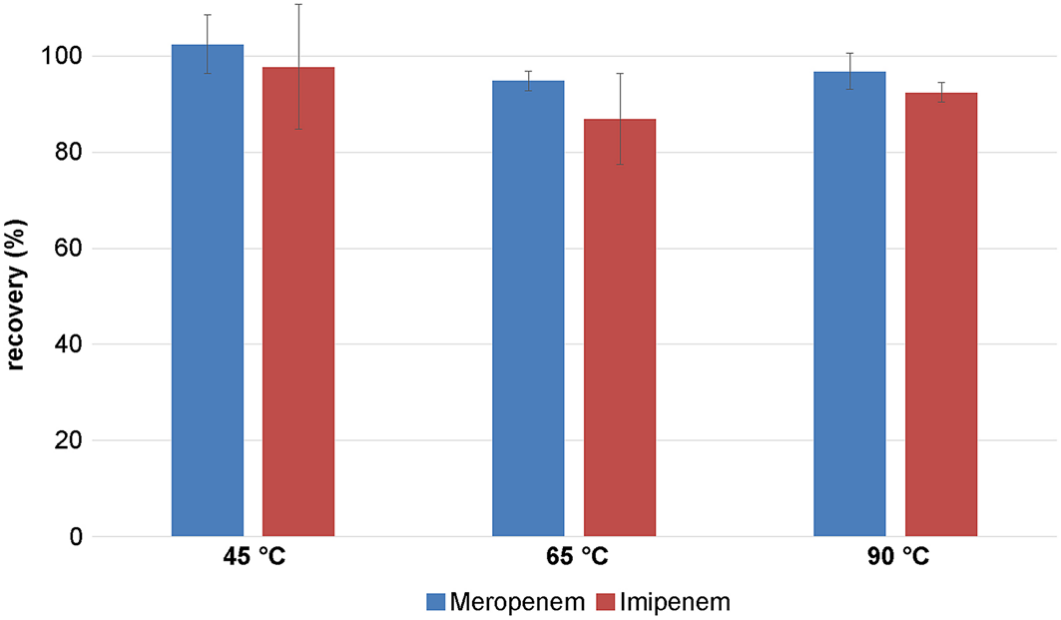
Recovery of temperature stressed meropenem and imipenem in powder after 120 min (*n* = 5)

Compared to the antibiotics in solution, the powder of meropenem and imipenem showed much lower degradation levels. Powdery meropenem and imipenem were tested at several temperatures (45 °C, 60 °C, 90 °C) after 120 min of heating (Fig. 7). Degradation levels of meropenem were about 5% at most and of imipenem 13% at most.

### Stability in PMMA cements after admixture

**Fig. 8:**
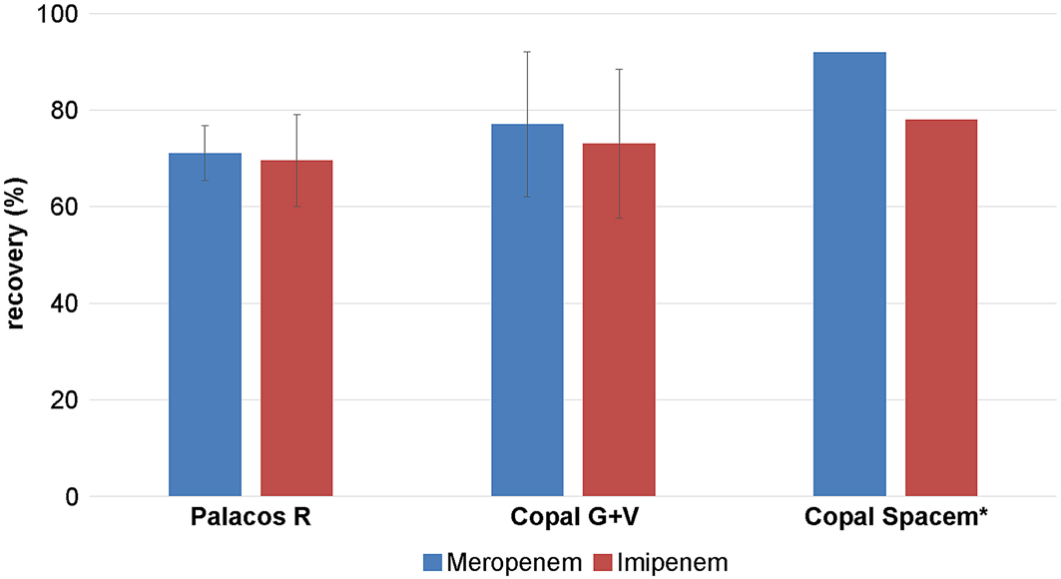
Recovery of 250 mg meropenem or imipenem after polymerization in three different PMMA bone cements. Palacos^®^ R: *n* = 4; Copal^®^ G + V: *n* = 3; Copal^®^ spacem: *n* = 1. * There is no standard deviation at Copal^®^ spacem because tests were performed only once

Stability tests of both carbapenems in bone cement showed that they remained largely stable during PMMA polymerization. Test series were performed with three different bone cements. Meropenem in a concentration of 250 mg in 15 g cement showed a degradation of 29% with Palacos^®^ R, 23% with Copal^®^ G + V and 8% with Copal^®^ spacem. Degradation levels of imipenem were 31% with Palacos^®^ R, 27% with Copal^®^ G + V and 22% with Copal^®^ spacem (Fig. 8).

**Fig. 9:**
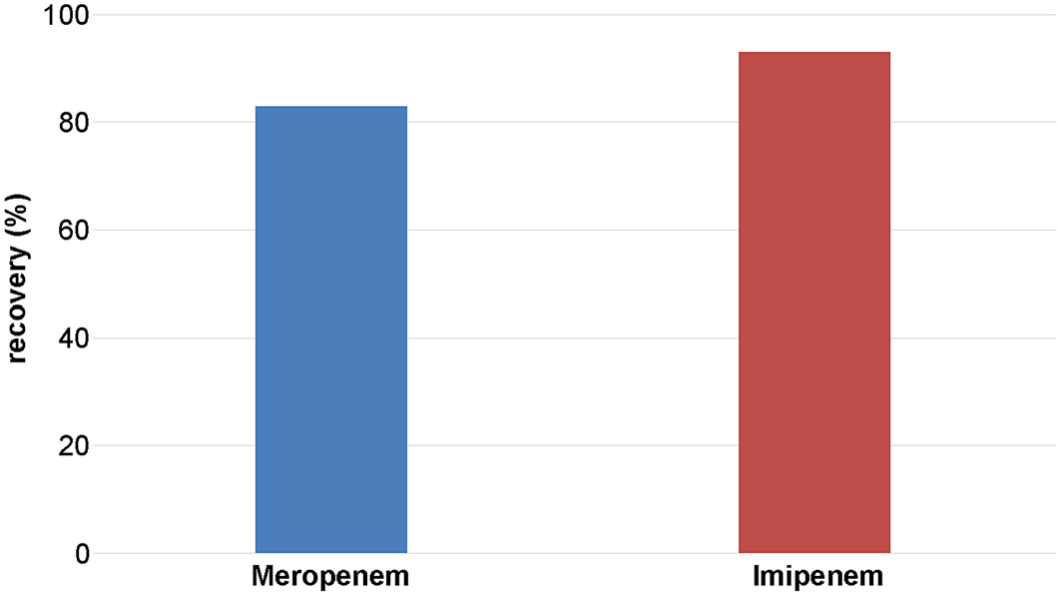
Degradation levels of 500 mg meropenem or imipenem after polymerization in PMMA Copal^®^ spacem. Copal^®^ spacem: *n* = 1

Further, the stability of 500 mg meropenem or imipenem in 30 g PMMA-cement was tested (Fig. 9). The bone cement used was Copal^®^ spacem, which showed a temperature maximum of 106 °C during the polymerization reaction. Meropenem showed a degradation level of 17%, and imipenem 7%.

### Temperature determination during polymerization reaction

**Fig. 10:**
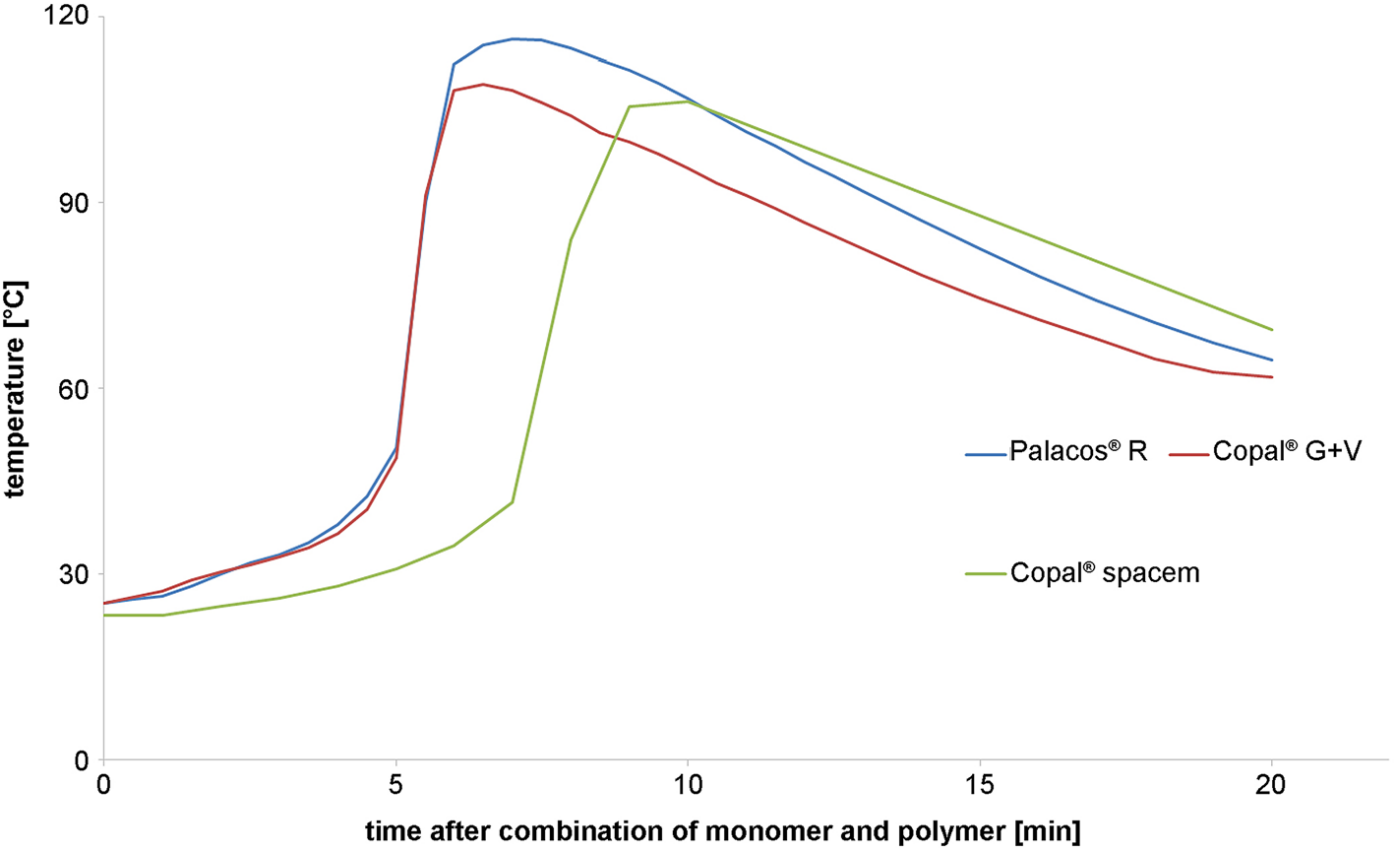
Reaction heat during polymerization of antibiotic-loaded PMMA bone cements

In addition to stability tests of meropenem and imipenem in PMMA bone cements, the heat development after combination of antibiotic-loaded polymer and liquid monomer was measured (Fig. 10). The highest detected temperature was 116 °C after about 7 min of polymerization for Palacos^®^ R.

## Discussion

Antibiotic-loaded bone cements are used in periprosthetic joint infections (PJI) to provide high local antibiotic concentrations. Since high polymerization temperatures can lead to degradation or inactivation of certain antibiotics, a key property for antibiotics used in combination with PMMA cements is thermal stability [21]. Decreased stability of β-lactam antibiotics in solution is already known. Even carbapenems such as meropenem and imipenem cannot resist degradation caused by the ring opening of the β-lactam ring via hydrolysis, which makes the substance useless in therapy [14].

Here, the stability of heat-stressed meropenem and imipenem under different conditions was investigated. Both antibiotics display similar pharmacokinetics, have good tissue penetration and express broad-spectrum bactericidal activity.

Samara et al. investigated, among others, the stability of meropenem and imipenem in aqueous solutions at body temperature (37 °C) over 42 days [22]. They found that both antibiotics decrease significantly over time, with in vitro degradation half-lives of 72 (± 3) days for imipenem and 67 (± 2.8) days for meropenem. Nevertheless, sustained antibacterial activity was observed for up to 3 weeks.

In the present study, exposure of carbapenems in liquid solution to higher temperatures led to higher degradation levels as well, and with increasing temperatures (65 to 90 °C), the degradation level was additionally affected by incubation time. However, temperature did hardly affect degradation levels if antibiotics were stressed in powder form for 120 min. An explanation for this observation could be that, during the preparation and hardening of the bone cement, only a limited amount of water is available that would promote hydrolysis.

Samara et al. further tested antibiotic stability in solution after a heat treatment that mimicked the curing of bone cement (Palacos^®^ R), with a temperature maximum of 83 °C for 1 min. Stability and antibacterial activity of imipenem, cilastatin and meropenem were not affected by the heat treatment compared to the non-treated group [22]. However, fast degradation levels detected by Samara et al. may be compromised by the carbapenems being maintained in aqueous solution, which does not accurately imitate conditions of antibiotics incorporated into PMMA cement. Since the present results showed that carbapenems in solid form are much more stable than in solution, it can be assumed that degradation in PMMA cements is probably not as fast.

In the present study, the stability of 250 mg meropenem and imipenem in 15 g PMMA bone cements was tested. Both antibiotics were relatively stable in bone cement, with retrieved amounts of about 70% in Palacos^®^ R and Copal^®^ G + V. Copal^®^ spacem showed a slightly better performance, especially in case of meropenem, with 90% retrieved antibiotic. Unfortunately, 15 g Copal^®^ spacem was tested only once, but similarly high amounts (80–90%) of antibiotic were detected when 500 mg antibiotic per 30 g cement was tested.

As seen in the present experiments and in contrast to the results of antibiotics in solution, meropenem and imipenem remain mostly stable in the cement after PMMA polymerization. The fair degradation levels of both antibiotics after exposure to temperatures < 120 °C allow the conclusion that they can potentially be used for an application in PMMA cements.

However, only 15 to 30 g cement was tested and higher amounts that are typically used in clinical practice may lead to increased polymerization temperatures. Báez et al. reported that meropenem-impregnated PMMA beads were not suitable for applications after autoclave sterilization, since zone growth inhibition was no longer observed after autoclaving [23]. They conclude that the exothermic reaction combined with the exposure to 121 °C for 15 min during autoclaving rendered meropenem incorporated into the cement bio-actively inert.

On the other hand, Baleani et al. reported that PMMA cements containing vancomycin and meropenem showed activity against bacteria that are unaffected by vancomycin, indicating an efficient elution of meropenem [24].

In fact, meropenem-loaded PMMA (Palacos^®^) has already been successfully used to treat human prosthetic joint infection, with the first report in 2010 [25]. Although in vivo studies are poor, meropenem-loaded PMMA cement is nowadays more often used for local antibiotic therapy [26].

The present results showed that imipenem was only slightly more affected by heating stress than meropenem under all investigated conditions. The degradation levels of imipenem and meropenem in PMMA cements were not significantly different, though. These findings suggest that imipenem might be a suitable candidate for local antibiotic therapy in cemented PJI as well.

Similar to meropenem, imipenem/cilastatin has been added in combination with vancomycin to PMMA cements [27]. Unfortunately, the in vitro study by Cerretani et al. was focused exclusively on the elution profile of vancomycin, reporting an increase of vancomycin elution when combined with imipenem. This phenomenon has been reported for the combination of vancomycin and meropenem too, it is called passive opportunism and is based on an increased porosity due to the addition of the second antibiotic. However, Cerretani et al. did neither analyze the elution profile of imipenem, nor did they conduct microbiologic studies on bactericidal efficacy against vancomycin-resistant but imipenem-sensitive germs. Thus, it is unclear if imipenem elution was effectively and if the antibiotic remained biologically active after admixture to the cement.

Among the tested cements, the degradation levels of both antibiotics appeared to be lower in Copal^®^ spacem. However, Copal^®^ spacem was tested only once under different conditions and further investigations are needed to determine the significance of the present results. Copal^®^ cement expresses superior elution profiles compared to Palacos^®^, which is inter alia related to a higher polymer-to-monomer ratio that leads to an increased porosity due to incomplete polymerization [28]. Thus, Copal^®^ cements might be better suited for combinations with meropenem and imipenem—not only based on the detected antibiotic recovery rates, but also because a more favorable elution profile is to be expected.

During polymerization, PMMA cements easily reach temperatures of 100 °C in vitro, which was confirmed by the present measurements. These high temperatures achieved in vitro are not necessarily met in vivo, where temperature is buffered by body fluids [22]. That means that the conditions for antibiotic use are different for one- and two-stage revisions. In case of one-stage revision, the antibiotic-loaded PMMA cement will cure inside the body, benefitting from the temperature buffering of the surrounding tissues. For two-stage revisions, spacers of various sizes are formed and cure outside the body, thereby being exposed to much higher temperatures, most likely resulting in higher degradation levels of meropenem and imipenem.

The present study has several limitations and an experimental setup closer to the real conditions during revision surgery (e.g., higher amount of PMMA cement and antibiotics, other form bodies) should be subject for further investigations. Unfortunately, it was not possible to include these conditions here, since only limited amounts of antibiotics and PMMA bone cement were available.

Furthermore, the elution profiles of meropenem and imipenem should be investigated over time, as well as the influence on mechanic characteristics of the cement. In vivo elution profile measurements would allow statements about the actual quantity of carbapenems delivered locally. More microbiologic studies on bactericidal efficacy against meropenem- and imipenem-sensitive germs are needed to investigate whether the elution is effectively and whether the antibiotic remains biologically active after admixture to the cement.

Supplementary, another interesting fact would be the temperature characteristics of bone cements in human body. In the present study, temperature of bone cements during polymerization was measured at room temperature, which means about 21 °C to 23 °C. Test series about temperature of bone cements in body core temperature, which is about 36 °C and 37 °C, could be substrate for continuative studies. Furthermore, as seen in the chromatographic results (Fig. 5) degradation products were observed during temperature stress tests, which will be subject to future investigations.

## Conclusions

Imipenem and meropenem are potential candidates for the admixture to PMMA bone cements deployed for local antibiotic therapy. Degradation levels after heat stress during polymerization reactions were acceptable and similar between both carbapenems. The best results were achieved in combination with Copal^®^ spacem. Further investigations are necessary to determine elution and bactericidal efficacy of the incorporated antibiotics.

## Data Availability

Data and material can be requested from the corresponding author.

## Abbreviations

G: Gentamicin
IPRP-HPLC DAD: Ion-pairing reversed-phase separation and diode array detection
OA: Osteoarthritis
PJI: Prosthetic joint infection
PMMA: Polymethyl methacrylate
V: Vancomycin
